# Mitogen-activated protein kinase p38-dependent phosphorylation of tristetraprolin enhances inflammatory and impairs antiviral responses to influenza virus

**DOI:** 10.1099/jgv.0.002360

**Published:** 2026-11-12

**Authors:** Prema Mono Nair, Yi Zhang, Malcolm R Starkey, Haw Tatt Jhong, Kamal Dua, Oliwia O Bolimowska, Alaina J Ammit, Andrew R Clark, Philip M Hansbro

**Affiliations:** 1 Priority Research Centres for Healthy Lungs and Grow Up Well, University of Newcastle and Hunter Medical Research Institute, Newcastle, New South Wales,, Australia; 2 School of Biomedical Sciences and Pharmacy, Faculty of Health and Medicine, University of Newcastle, Callaghan, New South Wales, Australia; 3 3 Department of Inflammation and Ageing, College of Medicine and Health, University of Birmingham, Birmingham, UK; 4 School of Medical Sciences, Faculty of Medicine and Health, The University of Sydney, Sydney, New South Wales, Australia; 5 Woolcock Emphysema Centre, Woolcock Institute of Medical Research, University of Sydney, Sydney, New South Wales, Australia; 6 School of Life Sciences, Faculty of Science, University of Technology Sydney, Sydney, New South Wales, Australia; 7 Woolcock Institute of Medical Research, Macquarie University, Sydney, New South Wales, Australia; 8 Centre for Inflammation, Centenary Institute and University of Technology Sydney, School of Life Sciences, Faculty of Science, Sydney, New South Wales, Australia

**Keywords:** cytokine storm, influenza A virus, inflammation, MAPK p38, tristetraprolin

## Abstract

In common with many other viruses, influenza A viruses (IAVs) activate the mitogen-activated protein kinase (MAPK) p38 pathway. This signalling pathway is strongly implicated in the pro-inflammatory cytokine storm that underlies morbidity and mortality in response to highly pathogenic IAV strains. We previously reported that MAPK p38 increased the expression of many inflammatory mediators via the phosphorylation and inactivation of the mRNA-destabilizing protein tristetraprolin (TTP). Hence, mice expressing a non-phosphorylatable form of endogenous TTP protein were strongly protected against inflammatory effects of polymicrobial sepsis or endotoxaemia. Here, we used the same minimally genetically modified mouse strain (*Zfp36aa/aa*) to investigate responses to a viral mimetic *in vitro* or IAV infection *in vivo*. When challenged *in vitro* with the viral mimetic poly(I:C), *Zfp36aa/aa* macrophages underexpressed several TTP-regulated inflammatory mediators. When infected with a pathogenic IAV strain, *Zfp36aa/aa* mice also under-expressed TTP-regulated inflammatory mediators. Strikingly, expression of type I and III IFNs and IFN-regulated antiviral genes was spared or enhanced in *Zfp36aa/aa* macrophages or mice. Consistent with these observations, *Zfp36aa/aa* mice cleared the pathogen more rapidly and were partially resistant to virus-induced airway pathology. These findings suggest that modulating TTP phosphorylation may exert anti-inflammatory effects whilst sparing or enhancing antiviral responses in the context of pathogenic IAV infection.

**Impact statement**

Nair and colleagues identify a mechanism promoting inflammatory responses to a pathogenic strain of influenza A virus (IAV). This mechanism may be a novel target for therapeutic strategies to minimize harmful cytokine storms, which cause illness and death in some individuals infected by IAV and other pathogenic viruses.

## Data summary

The authors confirm all supporting data, code and protocols have been provided within the article or through supplementary data files.

## Introduction

Seasonal influenza A virus (IAV) infection causes 3–5 million cases of severe illness and up to 650,000 deaths per year, largely amongst the elderly and immunocompromised [1]. An even greater threat to global health comes from IAV strains that naturally infect other organisms, such as birds or pigs, occasionally crossing species boundaries to infect humans. Because of sequence differences in viral surface antigens, existing immunity derived from vaccination or previous IAV exposure provides inadequate protection. Zoonotic outbreaks following escape of a virus from its usual host species are often associated with higher rates of morbidity and mortality, therefore, constituting major pandemic risks. For example, an avian strain of IAV, H1N1, gave rise to the flu pandemic of 1918–1919, which led to an estimated 50 million deaths worldwide [2]. Another IAV strain, H5N1, first crossed the species barrier from birds to humans in 2003 and has been associated with a fatality rate of up to 50% in outbreaks that were successfully contained. H5N1 and other variants of concern are continually monitored as potential sources of future pandemics [3].

In severe influenza, damage and dysfunction of airways is only partly due to direct effects of the virus, such as the death of infected airway epithelial cells. To a large extent, damage to airways and other tissues is driven by a dysregulated inflammatory response of the innate immune system, involving enhanced production of cytokines, chemokines and other inflammatory mediators, sometimes referred to as a cytokine storm. Although cytokine storms are reported in many virus-induced respiratory pathologies, the term lacks a precise and consistent definition [4]. Presentation differs according to pathogen, when and in what tissue or body fluid pro-inflammatory mediators are measured. Factors that have been frequently associated with IAV-induced cytokine storm include TNFα, IL6, IL-1β, CCL3, CCL4, CCL5, CXCL8, CXCL10 and the type I IFNs IFNα and IFNβ. Importantly, these all contribute to protection against viral pathogens; it is their excessive and dysregulated production that promotes pathogenesis in the context of severe respiratory disease. Neutrophils and macrophages derived from airway-infiltrating monocytes are strongly implicated in the excessive inflammatory response and tissue damage caused by pathogenic IAV strains [5–10].

Vaccination and the use of antiviral drugs can be effective strategies to prevent or mitigate IAV-induced respiratory disease. However, development and deployment of vaccines against novel strains is time-consuming. The short life-cycle of the virus, its rapid mutation rate and capacity for generation of novelty via reassortment of its segmented genome all favour the appearance of vaccine-resistant lines. The same features permit the emergence of strains that are resistant to antiviral drugs. In this light, there are strong arguments for the development of host-directed therapies, which target cellular pathways used by the virus to support infection, replication or release; aim to mitigate the harmful virus-induced cytokine storm; or both of the above [11].

Infected cells recognize IAV RNA via pattern recognition receptors (PRRs) of the TLR and RLR families (TLR is Toll-like receptor; RLR is RIG-I-like receptor; RIG-I is retinoic acid-inducible gene I) [12]. The signalling pathways activated in response to engagement of these PRRs include the mitogen-activated protein kinase (MAPK) p38 pathway. MAPK p38 activation is a stereotypical response to viral infection, which may be hijacked by viruses to promote their own propagation [13–15]. Accordingly, IAV propagation was reduced by pharmacological inhibition of MAPK p38 [16–23] or blockade of the pathway using siRNAs [24]. MAPK p38 inhibitors also reduced the expression of several pro-inflammatory mediators, suggesting that these drugs may have dual benefits in the treatment of virus-induced respiratory pathology [13].

MAPK p38-dependent expression of pro-inflammatory mediators in severe respiratory illness is usually attributed to dysregulation at the transcriptional level, although no unifying transcriptional mechanism has been established [25]. On the other hand, there is a credible, unifying post-transcriptional mechanism that has been documented for more than two decades [26, 27], but is often overlooked in the context of viral disease. TNF-α, IL-6, IL-1β and several other factors involved in the viral cytokine storm are negatively regulated by the mRNA-destabilizing factor tristetraprolin (TTP) [28]. MAPK p38-dependent phosphorylation of TTP protects it from proteasome-mediated degradation, but also inhibits its mRNA-destabilizing activity, causing an increase in expression of several inflammatory mediators [29, 30]. As MAPK p38 activity declines, TTP is dephosphorylated and activated. It then promotes the degradation of target mRNAs, contributing to the off-phase of the inflammatory response [27]. Sustained activation of MAPK p38 causes overexpression of inflammatory mediators in a manner that depends on TTP phosphorylation [31].

We previously generated a genetically modified mouse strain in which the two critical sites of MAPK p38-dependent phosphorylation of endogenous TTP protein, Ser52 and Ser178, were substituted by alanine residues. The formal name of the gene encoding TTP is *Zfp36* and the modified allele is known as *Zfp36aa*. In response to the pro-inflammatory stimulus LPS, the modified form of endogenous TTP protein (known as TTPaa) was weakly expressed by *Zfp36aa/aa* macrophages but functioned as a potent negative regulator of inflammatory responses, since it could not be phosphorylated and inactivated in a MAPK p38-dependent manner [30]. *Zfp36aa/aa* mice were protected against severe inflammation in experimental models of endotoxaemia, polymicrobial sepsis, cigarette smoke-induced airway inflammation and rheumatoid arthritis *Zfp36aa/aa* [30, 32–35]. In other words, the *Zfp36aa* mutation results in a notable gain-of-function of a key anti-inflammatory factor. We hypothesized that IAV induces (and benefits from) MAPK p38 activation, and that the cytokine storm is collateral damage caused at least in part by the consequent phosphorylation and inactivation of TTP.

## Methods

### Mouse macrophage isolation and culture

WT C57BL/6J and *Zfp36aa/aa* mice were housed at the University of Birmingham Biomedical Services unit, and all maintenance and procedures were carried out according to the Home Office guidelines and approved by the University of Birmingham Animal Welfare and Ethical Review Board. Bone marrow from *Ifnar1-/-* mice was generously provided by Professor Simon Arthur (University of Dundee). Mice were sacrificed by cervical dislocation, and legs were removed for bone marrow isolation. Femurs and tibiae were cleaned of muscle tissue and cut at each end. Bone marrow was extracted by centrifugation and plated in RPMI 1640 + l-glutamine (Gibco #21875034) supplemented with 10% heat-inactivated FCS (Sigma-Aldrich #F2442) and 50 ng ml^−1^ M-CSF (PeproTech #300–25) for 7 days to differentiate bone marrow-derived macrophages (BMDMs). According to flow cytometric analysis of macrophage cell surface markers, this isolation method yielded populations of primary macrophages that were >95% pure [36].

For stimulations, cells were seeded at 1×10^6^ cells ml^−1^ in tissue culture-treated 12-well or 6-well plates unless otherwise stated. Stimulation medium was prepared with LPS (*Escherichia coli*, Serotype EH100 (Ra) (TLRgrade™) – Enzo Life Sciences #ALX-581–010 L002) at 10 ng ml^−1^, recombinant human IFN-β (Peprotech 300-02BC) at 10 ng ml^−1^, or Poly(I:C) (High molecular weight) (InvivoGen # tlrl-pic) at 10 µg ml^−1^, without M-CSF. Ruxolitinib (Rux) (Stratech; A3012-APE) was added alongside the stimulating agents at 1 µM, with DMSO as a vehicle control. Where stated, murine macrophages were pre-incubated with IFNAR1 monoclonal antibody (Life Technologies; MAR1-5A3) or mouse IgG1-kappa isotype control (Thermo Fisher Scientific) for 2 h prior to stimulations. The supernatants and cell lysates were collected at relevant timepoints and stored at −80 °C.

### Western blot

Cells were lysed in radioimmunoprecipitation assay (RIPA) buffer (SDS-PAGE loading buffer in the case of p38/P-p38). Samples were passed through a QIAshredder column to remove genomic DNA (QIAGEN, 79656). If lysed in RIPA buffer, the protein was quantified by Pierce BCA Protein Assay (Thermo Fisher #23225), XT sample buffer+reducing agent or Laemmli loading buffer+β-mercaptoethanol was added, and an equal protein mass was loaded onto the gel. Before loading, samples were heated to 95 °C for 5 min. Western blotting was performed using XT Bis-Tris protein gels (Bio-Rad). Protein was transferred to Bio-Rad Trans-Blot polyvinylidene difluoride membranes (Bio-Rad, 1704157) using the Bio-Rad Trans-Blot Turbo transfer system. Primary antibodies were applied overnight at 4 °C as specified in Table 1. HRP-conjugated secondary antibodies were obtained from Cell Signalling Technologies (#7074, #7076) and applied for 1 h at room temperature. Imaging was performed using Clarity Enhanced Chemiluminescence substrate (Bio-Rad #1705061) and a ChemiDoc MP Imaging System (Bio-Rad). Densitometry was performed using ImageJ.

**Table 1.** Antibodies used for Western blotting

Protein	Supplier	Cat #	Host species	Species used for
Phospho-STAT1	Cell signalling	9167S	Rabbit	Human mouse
β-Actin	Merck	A1978	Mouse	Human mouse
TTP	Cell signalling	71632S	Rabbit	Human mouse
RIG-I	Cell signalling	4200S	Rabbit	Human mouse
Viperin	Abcam	AB107359	Mouse	Human mouse
β-Actin	Merck	A1978	Mouse	Human mouse

### Measurement of RNA

RNA was isolated using the Norgen Total RNA Purification Plus kit (Geneflow, P4-0016) according to the manufacturer’s protocol and quantified by NanoDrop (Thermo Fisher). cDNA was synthesized using iScript Reverse Transcriptase (Bio-Rad #1708891) from 250 ng RNA/reaction. Gene expression was measured by reverse transcription-quantitative polymerase chain reaction (RT-qPCR) using the Bio-Rad CFX384 system.

SYBR TB Green Premix Ex Taq (Takara, #RR820W) and primers supplied by Sigma-Aldrich (see Table 2) were used unless otherwise stated. UBC (human) or Rpl13a (mouse) were used to normalize mRNA measurements via the 2^−ΔΔCt^ method.

**Table 2.** RT-qPCR primer sequences for human and mouse

Gene name	Forward primer (5′→ 3′)	Reverse primer (5′→ 3′)
*B2m*	CTGCTACGTAACACAGTTCCACCC	CATGATGCTTGATCACATGTCTCG
*Tnf*	TCTGTCTACTGAACTTCGGGGTGA	TTGTCTTTGAGATCCATGCCGTT
*Cxcl1*	GCTGGGATTCACCTCAAGAA	CTTGGGGACACCTTTTAGCA
*Cxcl2*	ATAGCCACTCTCAAGGGCG	TCTCAGACAGCGAGGCACATC
*Ifnb1*	CCCTATGGAGATGACGGAGA	ACCCAGTGCTGGAGAAATTG
*Rsad2*	TGGCCGTGGTCAAGGAAAAA	TTAGGAGGCACTGGAAAACCTTC
*Ddx58*	GCTTTGGAGAAAGACAACAGCA	CACAACCAGTAGGGGCACAT
*Il1b*	TGCCACCTTTTGACAGTGATG	AAGGTCCACGGGAAAGACAC
*Ifnl1*	CTTCAGGCCACAGCAGAGCCCAAG	ACACACTTGAGGTCCCGGAGGA
*Tlr7*	AGTGCCTGAAAAATGCCCTG	GCTCTCTGAAGAATGTCACCAC
*Hprt*	AGGCCAGACTTTGTTGGATTTGAA	CAACTTGCGCTCATCTTAGGATTT

Whole lungs were collected and stored in RNA Stabilization Reagent, RNA*later* (Qiagen). RNA was extracted using guanidinium thiocyanate phenol chloroform (TRIzol) extraction. Extracted RNA was treated with DNase I (Sigma) and reverse-transcribed using BioScript (Bioline) and random hexamer primers (Invitrogen). The relative abundance of *Tnf*, *Cxcl1*, *Cxcl2*, *Il1b*, *Ifnb1*, *Ifnl1*, *Ddx58* and *Tlr7* mRNAs was determined relative to the reference gene *Hprt* by qPCR using a ViiA 7 Real-Time PCR System (Life Technologies).

### TTP Atlas

TTP Atlas is an online resource based on analysis of LPS-stimulated WT (*Zfp36+/+*) and TTP knockout (*Zfp36-/*-) mouse BMDMs [37] (https://ttp-atlas.univie.ac.at). It provides ChIP-seq data precisely mapping sites of interaction with mRNA; RNA-seq data quantifying steady-state mRNA expression at 0, 3, 6 and 9 h after addition of LPS; and estimates of RNA half-life at 3, 6 and 9 h after addition of LPS. TTP is a very well-characterized negative regulator of inflammatory gene expression, which functions by binding to 3′ UTRs of target mRNAs. Several instances of intronic binding have also been documented, but these are not associated with changes in target gene expression. In the majority of cases, the binding of TTP to the 3′ UTR of an mRNA results in both reduced stability and decreased steady-state expression levels. In TTP Atlas, authentic TTP targets are characterized by (i) robust TTP binding within the 3′ UTR, as analysed using a stringent peak-finding algorithm; (ii) increased mRNA stability in *Zfp36-/-* macrophages; (iii) increased steady-state mRNA expression in *Zfp36-/-* macrophages. We used these criteria to determine whether mRNAs encoding antiviral mediators are targeted by TTP.

### Influenza virus infection

Male, 6–9 weeks old, *Zfp36*^+/+^ or *Zfp36^aa/aa^* C57BL/6 mice were anaesthetized with isoflurane and infected intranasally (i.n.) with 33 p.f.u. of the mouse-adapted H1N1 influenza virus A/PR/8/34 (WHO Collaborating Centre for Reference and Research of Influenza, Victoria, Australia) in 50 µl of medium (UltraMDCK, Lonza). Controls were sham-inoculated with medium. Mice were sacrificed at 1, 3, 5, 7 and 9 days post-infection.

### Viral titre

Madin-Darby Canine Kidney cells were grown until ∼79% confluent. Cells were then washed with Dulbecco’s Phosphate Buffered Saline (Sigma) three times and submerged in Leibovitz’s L-15 (L-15) medium (Invitrogen) supplemented with HEPES (Invitrogen) and N-p-tosyl-l-phenylalanine chloromethyl ketone-treated trypsin (trypsin-TPCK; Invitrogen). Bronchoalveolar lavage (BAL) fluid samples were serially diluted in L-15 medium supplemented with HEPES and added to the cells. Inoculum was removed after 60 min of incubation (37 °C with 5% CO_2_). Next, a thin overlay of 1.8% agarose in L-15 medium containing trypsin-TPCK (Invitrogen) was placed onto the cell monolayers. Plaques were stained with 0.1% crystal violet and counted after 48 h of incubation (37 °C with 5% CO_2_) [38].

### Airway inflammation

BAL was performed; cells were cytocentrifuged (300×*g*, 10 min) stained with May–Grunwald–Giemsa and differential leucocyte counts were determined according to morphological criteria from a total of 250 cells as previously described [39].

### Protein isolation

Proteins were extracted from whole lungs. Briefly, tissues were homogenized in 500 µl of sterile Dulbecco’s phosphate-buffered saline (Life Technologies) supplemented with PhoSTOP phosphatase and Complete ULTRA protease inhibitor cocktails (Roche Diagnostics) using a Tissue Tearor stick homogenizer (BioSpec Products) on ice. Homogenates were then centrifuged (8,000*×g*, 10 min, 4 °C). Supernatants were collected and stored at −20 °C for assessment by ELISA.

### Histopathology

Lungs were formalin-fixed, embedded and sectioned. Longitudinal sections were stained with H and E. Histopathology was scored in a blinded fashion according to a set of custom-designed criteria (Table 3).

**Table 3.** Histopathological scoring system

**Score 1: Airways inflammation score (/4**) 0=Lack of inflammatory cells around airways – Absent 1=Some airways have small numbers of inflammatory cells – Mild 2=Some airways have significant inflammation – Moderate 3=Majority of airways have some inflammation – Marked 4=Majority of airways have significant inflammation – Severe
**Score 2: Vascular inflammation score (/4**) 0=Lack of inflammatory cells around blood vessels – Absent 1=Some blood vessels have small numbers of inflammatory cells – Mild 2=Some blood vessels have significant inflammation – Moderate 3=Majority of blood vessels have some inflammation – Marked 4=Majority of blood vessels have significant inflammation – Severe
**Score 2: Parenchymal inflammation (at 10× magnification) (/5**) 0= <1% affected 1=1–9% affected 2=10–29% affected 3=30–49% affected 4=50–69% affected 5= >70% affected
**Total score=Score 1+Score 2+Score 3**

### Airway remodelling

Formalin-fixed, H and E-stained longitudinal lung sections were assessed for small airway epithelial thickening as described [34]. For a minimum of four small airways (<1,000 µm perimeter) per section, the ratio of epithelial cell area (μm^2^) to basement membrane (BM) perimeter (μm) was calculated.

### Cytokine and chemokine concentrations in lung

A multi-analyte flow assay kit (BioLegend) was used according to the manufacturer’s instructions to measure concentrations in lung homogenates of TNFα, CXCL1, CCL3, IL6 and CXCL10.

## Results

### Gain-of-function mutation of the *Zfp36* (TTP) gene differentially affects the expression of pro-inflammatory and anti-viral genes in activated primary macrophages

In a previously reported experiment [31], the expression of several pro-inflammatory mediators was significantly lower in LPS-treated *Zfp36aa/aa* macrophages than in LPS-treated WT control macrophages. In the same experiment, many antiviral factors were induced by LPS, but their expression was relatively delayed and unaffected by the *Zfp36* gene modification (Supplemental Fig. 1). As a representative example, Fig. 1A illustrates the expression of *Rsad2* mRNA, which encodes the broad-action antiviral factor Viperin [40]. Based on several lines of evidence, the expression of antiviral mediators was dependent on autocrine type I IFN-mediated signalling. Again used as a representative example, *Rsad2* expression was similarly induced by LPS and IFNβ (Fig. 1B). LPS-induced expression of *Rsad2* was significantly impaired by the JAK inhibitor Rux, which blocks signalling by JAK-associated receptors, including the type I IFN receptor IFNAR (Fig. 1C). LPS itself does not directly activate JAKs, therefore, JAK-dependent responses are assumed to be mediated by LPS-induced secreted factors. LPS-induced expression of *Rsad2* was significantly inhibited by a neutralizing antibody against IFNAR (Fig. 1D). LPS-induced expression of *Rsad2* was ablated in *Ifnar1-/-* macrophages lacking a functional type I IFN receptor (Fig. 1E). Several other antiviral factors behaved very similarly in these experiments (data not shown). It was confirmed that both Rux and the IFNAR-neutralizing antibody interrupted LPS-induced STAT1 phosphorylation, which is critical for the expression of antiviral mediators (Fig. 1F).

**Fig. 1.**
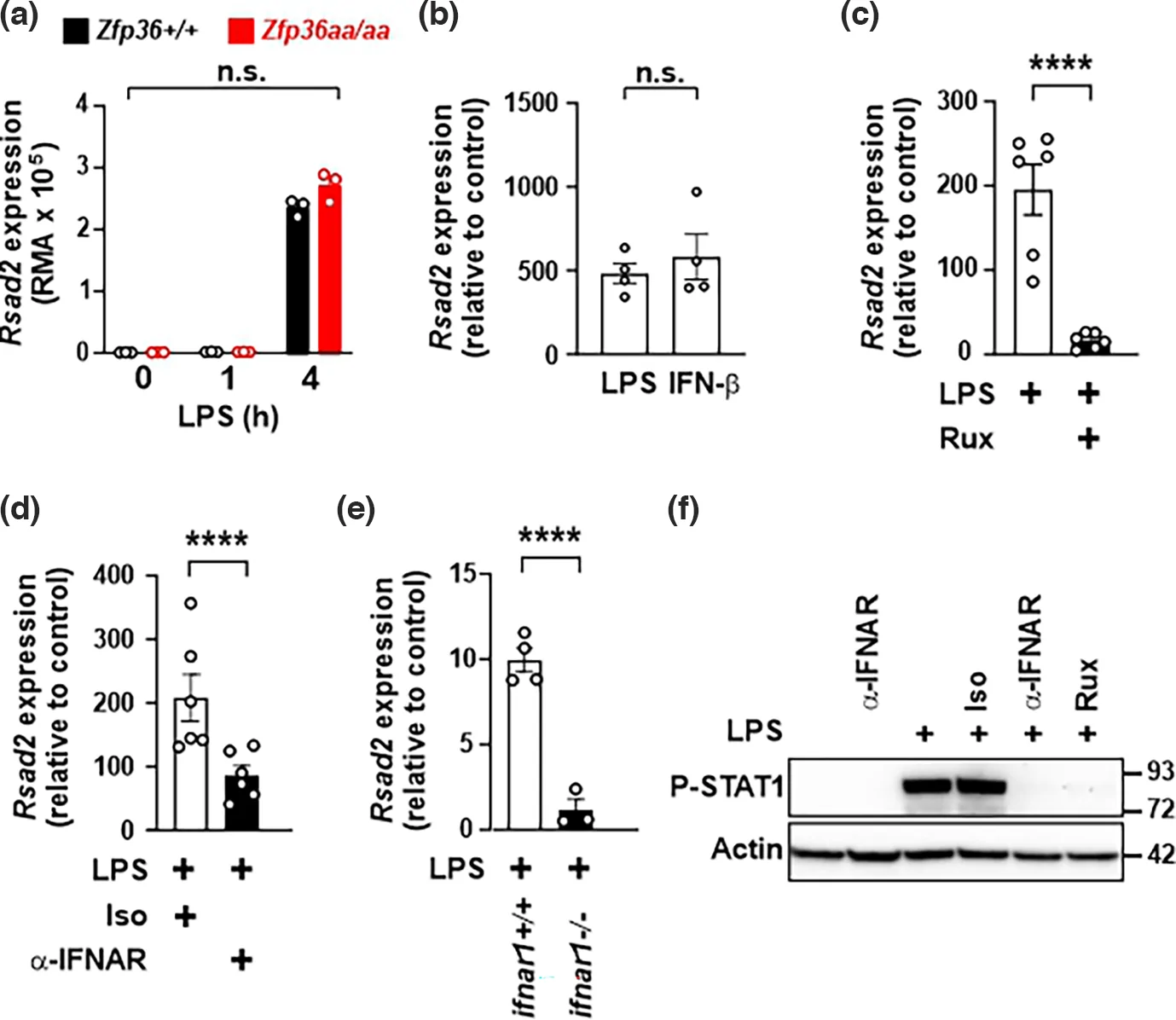
LPS-induced expression of type I IFN-dependent antiviral genes is spared in *Zfp36aa/aa* BMDMs. (**a**) LPS-induced expression of *Rsad2* mRNA does not differ between *Zfp36+/+* + and *Zfp36aa/aa* BMDMs. Microarray data from a previously reported experiment [31]. (**b-e**) *Rsad2* mRNA was measured by quantitative PCR and fold changes of gene expression were calculated by ΔΔCt method with normalization against *B2m* mRNA and unstimulated controls. (**b**) WT BMDMs were treated for 4 h with LPS (10 ng ml^−1^) or IFN-β (10 ng ml^−1^). Mean±sem fold change of *Rsad2* mRNA from four independent repeats. (**c**) WT BMDMs were treated with LPS (10 ng ml^−1^) for 4 h in the presence of 1 µM Rux (Rux) or vehicle control (0.1% DMSO). Mean±sem fold change of *Rsad2* mRNA from six independent repeats. (**d**) WT BMDMs were treated with LPS (10 ng ml^−1^) in the presence of an IFNAR-blocking antibody or isotype control (each 10 µg ml^−1^). Mean±sem fold change of *Rsad2* mRNA from six independent repeats. (**e**) *Ifnar1+/+* + or *Ifnar1-/-* BMDMs were generated from cryopreserved bone marrow and stimulated for 4 h with LPS (10 ng ml^−1^). Mean±sem fold change of *Rsad2* mRNA from four (*Ifnar+/+*) or three (*Ifnar-/-*) independent repeats. (**f**) WT BMDMs were treated with LPS (10 ng ml^−1^) for 4 h in the presence of an IFNAR-blocking antibody, isotype control (each 10 µg ml^−1^) or 1 µM Rux. Phosphorylated STAT1 (P-STAT1) and actin were detected by Western blotting of whole-cell lysates. Representative of three independent repeats. n.s., *P*>0.05; ****P<0.001. Unpaired two-tailed t-test.

We arbitrarily selected 40 ISGs that were strongly induced by LPS in mouse macrophages and used TTP Atlas (https://ttp-atlas.univie.ac.at) [37] to look for evidence of direct regulation by TTP (see Methods). Authentic, well-characterized TTP targets, such as *Tnf*, *Cxcl1*, *Cxcl2*, *Ier3* and *Il10* all demonstrated clear binding of TTP in their 3′ UTRs, increased mRNA stability and enhanced mRNA expression in TTP knockout (*Zfp36-/-*) macrophages in this database. No ISGs displayed the same characteristics. The majority of ISG mRNAs were stable. A few displayed intronic binding of TTP but no increase of stability or steady-state expression in *Zfp36-/-* macrophages. Of the surveyed mRNAs only two (*Ifit2* and *Usp18*) demonstrated TTP binding in their 3′ UTRs and increased expression in *Zfp36-/-* macrophages. However, both of these mRNAs were stable, therefore, the effects of *Zfp36* gene disruption on their expression may be indirect. These findings suggest that TTP does not directly regulate the expression of most ISGs, as one might have predicted. The dynamic regulation of TTP function supports transient expression of pro-inflammatory mediators [27]. In contrast, the IFN-I-dependent antiviral state needs to be long-lasting to provide effective protection.

To extend these observations to a stimulus more directly relevant to viral challenges, *Zfp36+/+* + and *Zfp36aa/aa* BMDMs were challenged with poly(I:C), a mimetic of viral dsRNA. Poly(I:C)-induced activation of MAPK p38 did not differ between *Zfp36+/+* + and *Zfp36aa/aa* BMDMs, peaking at 1 h then declining (Fig. 2A). In WT BMDMs poly(I:C) induced both expression and phosphorylation of TTP (phospho-TTP migrates with lower electrophoretic mobility and is indicated by the asterisk in Fig. 2B). Poly(I:C)-induced expression and phosphorylation of TTP were reduced in *Zfp36aa/aa* BMDMs. This is reminiscent of the MAPK p38-dependent phosphorylation and stabilization of TTP in response to treatment of macrophages with LPS [29, 30, 41]. Others have described a MAPK p38-dependent increase in TTP protein expression and phosphorylation in response to poly(I:C) in a human monocyte/macrophage cell line [42].

**Fig. 2.**
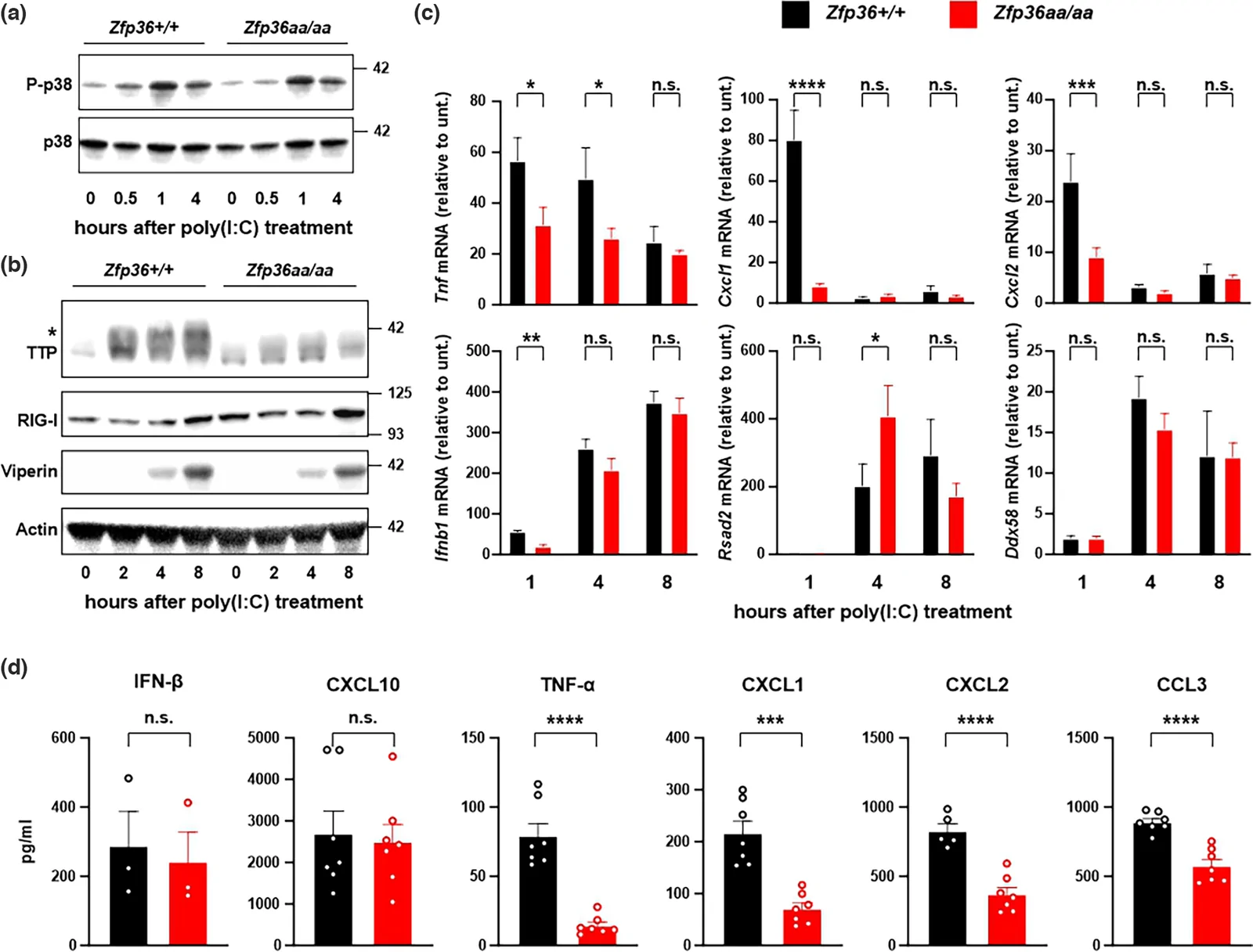
TTP phosphorylation differentially affects pro-inflammatory and antiviral gene expression in response to poly(I:C). (**a**) *Zfp36+/+* + or *Zfp36aa/aa* BMDMs were stimulated for 0.5, 1 or 4 h with 10 µg ml^−1^ poly(I:C). Total and phosphorylated (activated) p38 were detected by Western blotting of whole-cell lysates. Representative of four independent experiments. (**b**) *Zfp36+/+* + or *Zfp36aa/aa* BMDMs were stimulated for 2, 4 or 8 h with 10 µg ml^−1^ poly(I:C). TTP, RIG-I, Viperin and actin were detected by Western blotting of whole-cell lysates. * indicates the position of migration of hyperphosphorylated TTP. Representative of at least three independent repeats. (**c**) *Zfp36+/+* + or *Zfp36aa/aa* BMDMs were stimulated for 1, 4 or 8 h with 10 µg ml^−1^ poly(I:C). Expression of selected mRNAs was assessed by quantitative PCR (ΔΔCt method with normalization against *B2m* mRNA and unstimulated control). Graphs show mean±sem fold change relative to control. Three to six independent repeats. n.s., *P*>0.05; *P<0.05; ***P<0.005; ****P<0.001. Two-way ANOVA with Tukey’s correction for multiple comparison. (**d**) *Zfp36+/+* + or *Zfp36aa/aa* BMDMs were stimulated for 8 h with 10 µg ml^−1^ poly(I:C). Concentrations of cytokines and chemokines in supernatants were measured by multiplexed cytometric bead assays. Graphs show mean±sem concentrations. Three to seven independent repeats. n.s., *P*>0.05; ***P<0.005; ****P<0.001. Unpaired two-way t-test.

Expression of viperin protein (encoded by *Rsad2* mRNA) was strongly induced by poly(I:C), and this response did not differ between *Zfp36+/+* + and *Zfp36aa/aa* BMDMs (Fig. 2B). Expression of RIG-I protein (encoded by *Ddx58* mRNA) was less strongly induced by poly(I:C). Again, this response was similar in *Zfp36+/+* + and *Zfp36aa/aa* BMDMs (Fig. 2B). The poly(I:C)-induced expression of TTP target mRNAs *Tnf*, *Cxcl1* and *Cxcl2* was significantly impaired in *Zfp36aa/aa* BMDMs (Fig. 2C). We previously reported that expression of *Ifnb1* mRNA (encoding IFN-β) was negatively regulated by TTP in LPS-stimulated BMDMs [43]. Consistent with this, *Ifnb1* mRNA was significantly under-expressed by poly(I:C) treated *Zfp36aa/aa* BMDMs, but only at the 1 h time point, when expression was still relatively weak. *Ifnb1* gene expression continued to increase strongly at 4 and 8 h and was not significantly altered in *Zfp36aa/aa* BMDMs at these later time points. Poly(I:C) strongly increased *Rsad2* mRNA expression in a manner that was not sensitive to TTP phosphorylation and increased *Ddx58* mRNA expression less strongly, but also independently of TTP phosphorylation (Fig. 2C). These results are consistent with the western blots shown above (Fig. 2B). Multiplex bead assays demonstrated similar expression of IFN-β and the type I IFN-dependent chemokine CXCL10 in poly(I:C)-treated Zfp36+/+ and *Zfp36aa/aa* BMDMs, whereas the expression of the TTP-regulated factors TNFα, CXCL1, CXCL2 and CCL3 was significantly impaired in *Zfp36aa/aa* BMDMs (Fig. 2D). Collectively, these results suggest that TTP differentially regulates pro-inflammatory and antiviral responses to the viral double-stranded RNA mimetic poly(I:C). Although the expression of *Ifnb1* mRNA may be sensitive to TTP during the early response, this does not seem to impair the expression of antiviral factors.

### *Zfp36aa/aa* mice are partially protected from harmful consequences of inoculation with a pathogen IAV strain

An obvious implication is that *Zfp36aa/aa* mice may be protected from inflammatory consequences of viral infection, much as they are protected from inflammatory pathology in experimental models of endotoxaemia and polymicrobial sepsis [30, 35, 44]. To test this hypothesis, *Zfp36+/+* + and *Zfp36aa/aa* mice were infected intranasally with IAV H1N1 strain A/PR/8/34 (hereafter referred to as H1N1), which is pathogenic in mice. We hypothesized that the targeted mutation of the *Zfp36* gene would influence the resolution of IAV-induced inflammation, therefore, we used a sub-lethal inoculum of H1N1. In whole lung homogenates, the expression of *Tnf*, *Cxcl1*, *Cxcl2* and *Il1b* mRNAs was increased by H1N1 infection as compared with sham infection, and these responses were impaired in *Zfp36aa/aa* mice (Fig. 3A, upper). In contrast, the virus-induced expression of *Ifnb1* and *Ifnl1* mRNAs was significantly increased in *Zfp36aa/aa* mice (Fig. 3A, lower). Expression of representative ISGs *Ddx58* and *Tlr3* did not differ between *Zfp36+/+* + and *Zfp36aa/aa* mice (Fig. 3A, lower). Of the cytokines/chemokines that could be detected in lung homogenates of H1N1-infected mice, TNF-α, CCL3 and IL6 levels were lower in *Zfp36aa/aa* mice (Fig. 3B).

**Fig. 3.**
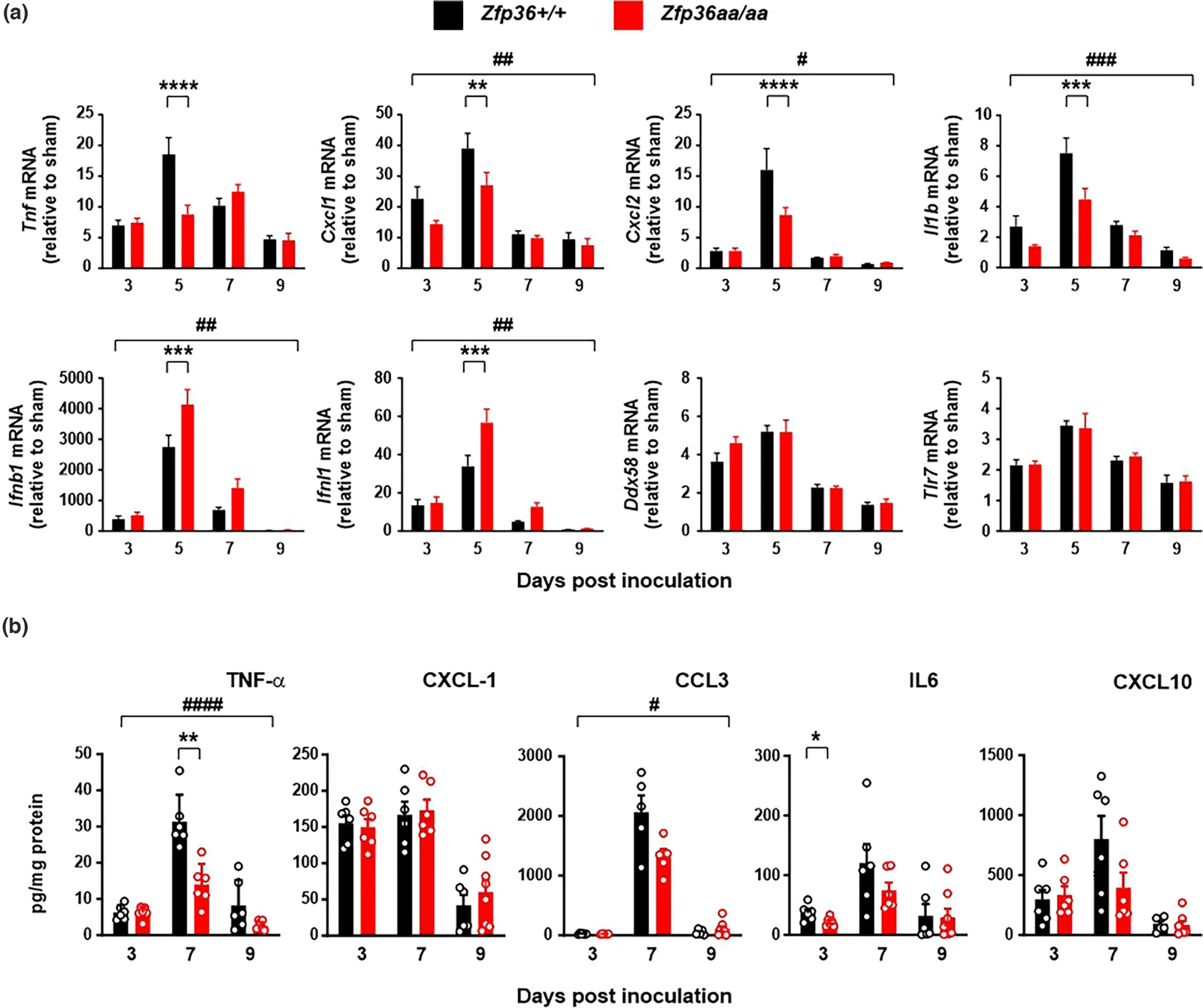
IAV-infected *Zfp36aa/aa* mice display lower expression of pro-inflammatory mediators but enhanced or unchanged expression of IFNs and IFN-regulated genes. (**a**) *Zfp36+/+* + or *Zfp36aa/aa* mice were sham inoculated or inoculated intranasally with 33 p.f.u.s of IAV A/PR/8/34. After 3, 5, 7 or 9 days mice were culled and selected mRNAs in whole lung homogenates were measured by quantitative PCR (ΔΔCt method with normalization against *Hprt* mRNA and sham-inoculated control day 0). Graphs show mean±sem increase, *n*=5–8. None of the mRNAs changed significantly over time in sham-inoculated animals, and these data are omitted for clarity. **P<0.01; ***P<0.005; ****P<0.001 (differences at individual time points). *P<0.05; **P<0.01; ***P<0.005 (effect of genotype across the entire time course). Comparisons that were not significantly different are not indicated. Two-way ANOVA with Tukey’s correction. (**b**) *Zfp36+/+* + or *Zfp36aa/aa* were treated as in (**a**). Cytokine or chemokine concentrations in whole lung homogenates were measured by cytometric bead assay. Graphs show mean±sem concentrations, *n*=6–8. n.s., *P*>0.05; *P<0.05; **P<0.01 (differences at individual time points). *P<0.05; ****P<0.0001 (effect of genotype across the entire time course). Comparisons that were not significantly different are not indicated. Two-way ANOVA with Tukey’s correction.

H1N1 caused leukocytic infiltration of mouse lungs, which was dominated by early recruitment of neutrophils and macrophages, peaking at day 3 after infection. Lymphocytes were recruited in lower numbers and later in the time course (peaking at day 9) (Fig. 4A). Total leucocyte numbers in BAL fluid were significantly lower in *Zfp36aa/aa* mice. This was mainly a result of impaired neutrophil recruitment, although leucocyte and macrophage numbers were also significantly reduced at day 9 (Fig. 4A). In a composite score of lung histopathology comprised of airway, vacuolar and parenchymal inflammation components, histopathology was lower in *Zfp36aa/aa* mice at day 9 post-infection (Fig. 4B and C). In both *Zfp36+/+* + and *Zfp36aa/aa* mice, H1N1 caused airway remodelling, manifested as thickening of airway epithelia. In *Zfp36aa/aa* but not *Zfp36+/+* + mice this response was reversed by day 9 post-infection (Fig. 4D). *Zfp36aa/aa* mice were also significantly protected against virus-induced weight loss, which is indicative of systemic inflammation (Fig. 5A). Finally, BAL fluid H1N1 viral titres were significantly reduced in *Zfp36aa/aa* mice, reflecting rapid clearance of the pathogen (Fig. 5B). Overall viral titres in this experiment were comparatively low, reflecting the sub-lethal inoculum and the fact that titres were measured in BAL fluid rather than whole-lung homogenates.

**Fig. 4.**
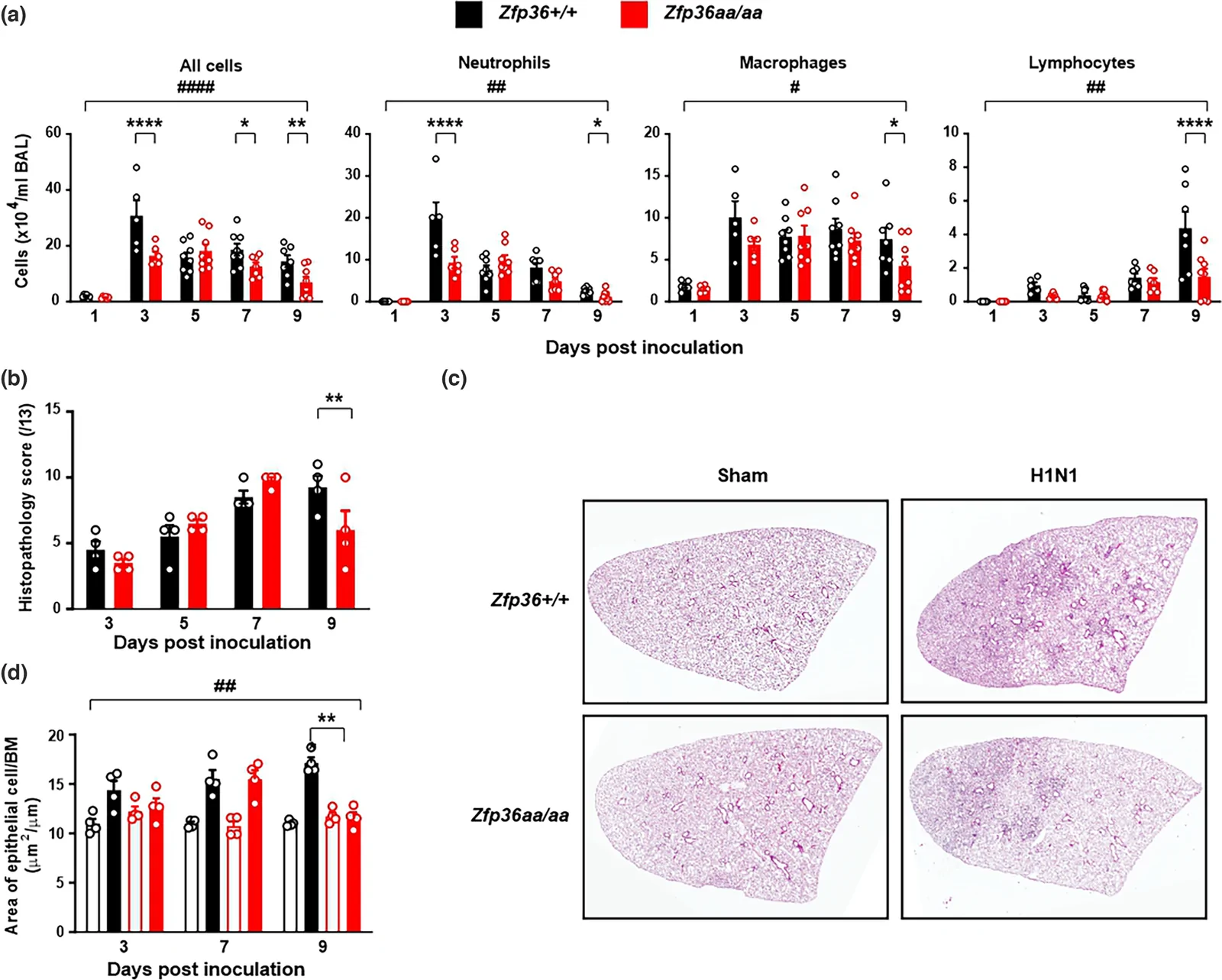
*Zfp36aa/aa* mice are partially protected from IAV-induced lung inflammation. *Zfp36+/+* + or *Zfp36aa/aa* mice were sham inoculated or inoculated intranasally with 33 p.f.u.s of IAV A/PR/8/34. (**a**) Populations of infiltrating cells in BAL fluid were counted 1, 3, 5, 7 or 9 days after inoculation. Numbers of cells in sham-inoculated mice were negligible at all time points, and these data are omitted for simplicity of presentation. Graphs show mean±sem total cells, neutrophils, macrophages or lymphocytes, five to eight mice per group. (**b**) Lungs were harvested and H and E-stained sections were scored for histopathology on a 13-point scale comprising airway, vascular and parenchymal elements. Individual element scores are shown in Fig. S2. Graphs show mean±sem total score, three mice per group. (**c**) Representative H and E-stained lung sections from day 9 post-inoculation. (**d**) Airway remodelling was assessed as epithelial cell area of small airways relative to BM perimeter. Graphs show mean±sem epithelial thickness, four mice per group. *P<0.05; **P<0.01; ****P<0.001 (differences at individual time points). *P<0.05; **P<0.01; ****P<0.001 (effect of genotype across the entire time course). Two-way ANOVA with Tukey’s correction.

**Fig. 5.**
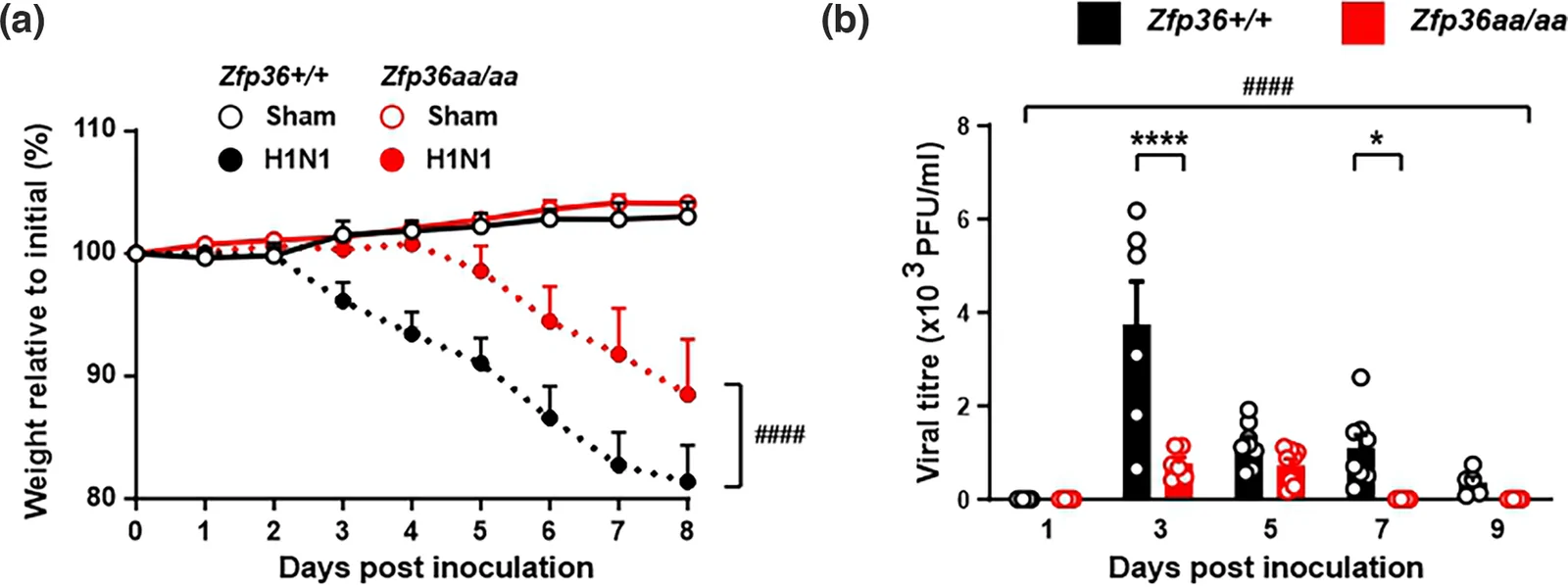
Weight loss and viral titre are reduced in IAV-inoculated Zfp36aa/aa mice. (**a**) *Zfp36+/+* + or *Zfp36aa/aa* mice were sham inoculated or inoculated with 33 p.f.u.s of IAV A/PR/8/34 and weight was measured over eight subsequent days. Six to eight mice per group. ****P<0.001 (effect of genotype over entire time course). Two-way ANOVA with Geisser–Greenhouse correction. (**b**) *Zfp36+/+* + or *Zfp36aa/aa* mice were sham inoculated or inoculated with 33 p.f.u.s of IAV A/PR/8/34 and viral titres in BAL fluid were measured at days 1, 3, 5, 7 or 9 post-inoculation. Graph shows mean±sem p.f.u. ml^−1^, five to eight animals per group. *P<0.05; ****P<0.001 (differences at individual time points). ****P<0.001 (effect of genotype over entire time course). Two-way ANOVA with Tukey’s correction.

## Discussion

The inflammatory component of the innate immune response to respiratory pathogens is necessary for rapid pathogen elimination and for engagement of the adaptive immune system to provide longer-term protection. Yet an excessive inflammatory response risks damaging the host and, paradoxically, may favour propagation of the pathogen [45]. Small differences in the magnitude or dynamics of the inflammatory response may tip the delicate balance towards effective pathogen clearance and resolution or towards immune-mediated tissue damage leading to morbidity or mortality. Here, we show that a minor genetic modification (substitution of two codons of an endogenous protein-coding gene) reduces the pathogenic consequences of IAV infection. *Zfp36aa/aa* mice were dramatically (although not completely) protected against infection-induced weight loss (Fig. 5A). They also demonstrated lower lung histopathology scores and reduced airway epithelial thickening (Fig. 4B–D). In IAV-infected *Zfp36aa/aa* mice, there was less infiltration of lungs by immune cells, particularly neutrophils (Fig. 4A). This is consistent with the previously reported role of TTP in regulating neutrophil migration by constraining the expression of neutrophil chemokines CXCL1 and CXCL2 [30, 32, 34, 46, 47] and the role of neutrophils in lung damage [48]. It should be noted that, whilst *Cxcl1* mRNA and CXCL1 protein were under-expressed by poly(I:C)-stimulated *Zfp36aa/aa* BMDMs (Fig. 2), only the mRNA displayed lower expression in infected *Zfp36aa/aa* mice, CXCL1 protein levels being similar in WT and *Zfp36aa/aa* lungs (Fig. 3). It is possible that TTP influences CXCL1 protein expression earlier in the course of IAV infection, which would have been missed by our experimental design. Expression of *Tnf* mRNA and TNFα protein was clearly reduced in both poly(I:C)-treated *Zfp36aa/aa* BMDMs and IAV-infected *Zfp36aa/aa* mice (Figs. 2 and 3). It is likely that the reduction in TNFα levels in *Zfp36aa/aa* mice contributed to protection against virus-induced pathology. Changes in expression of other TTP-regulated factors, including CCL3, IL-6 and IL-1β may also have contributed to this outcome. These findings indicate that activation of the MAPK p38 pathway, a common response to respiratory viruses [13], drives expression of inflammatory mediators at least partly via the phosphorylation and inactivation of TTP.

*Ifnb1* mRNA was over-expressed by LPS-treated *Zfp36-/-* BMDMs [37] and under-expressed by LPS-treated *Zfp36aa/aa* BMDMs [43], consistent with its negative regulation by TTP. Contrastingly, in the present study, *in vitro* expression of *Ifnb1* mRNA in response to poly(I:C) was only transiently affected by the *Zfp36aa* mutation (Figs. 2C and 1 h time point), and the expression of IFN-β protein was not significantly affected (Fig. 2D). The time courses of *Ifnb1* expression differ markedly between the two stimuli. Expression in response to LPS is extremely transient, peaking at 2 h, whereas expression in response to poly(I:C) is very sustained, still increasing at 12 h [43]. We speculate that the very transient activation of *Ifnb1* transcription by LPS [49] makes gene expression highly sensitive to modulation of mRNA stability, whereas sustained transcriptional activation by poly(I:C) renders *Ifnb1* gene expression less sensitive to post-transcriptional regulation. This hypothesis is under investigation. The situation *in vivo* is yet more complex because of changing viral loads and cellular populations in the lungs of infected animals.

Importantly, the expression of IFN-stimulated/antiviral genes appeared to be spared from negative regulation by TTP both *in vitro* and *in vivo*. This makes evolutionary sense because the antiviral state needs to be durable rather than transient. It is also consistent with reports that MAPK p38 inhibitors reduced expression of inflammatory mediators but spared or enhanced the expression of antiviral genes in respiratory virus-infected cells [14, 50]. Furthermore, *Zfp36* is itself an IFN-stimulated gene [51, 52] that may contribute to antiviral defences either by targeting viral RNA for degradation [53, 54] or by supporting the function of RIG-I, a critical sensor of viral nucleic acids [55]. Like RIG-I, TTP localizes to stress granules, which are hubs for detection of viruses and initiation of antiviral responses [56]: this localization is impaired by MAPK p38-dependent phosphorylation of TTP [57]. It is notable that expression of both *Ifnb1* and *Ifnl1* mRNAs was increased in lungs of IAV-infected *Zfp36aa/aa* mice (Fig. 3A), and that the virus was significantly more rapidly cleared by *Zfp36aa/aa* mice (Fig. 5B). There are at least two possible explanations for the enhanced viral clearance by *Zfp36aa/aa* mice. The first is that the inflammatory response is counterproductive in this experimental model, favouring viral replication such that a decrease in inflammation and inflammatory cell recruitment accelerates clearance. The second is that TTP has some as yet ill-defined antiviral role that is impaired by its phosphorylation. In principle, this would provide an additional benefit to viruses from the sustained activation of MAPK p38. We are currently investigating both hypotheses, which are not mutually exclusive.

The relationship between viral load and host gene expression in the lung is complex and bi-directional. We have interpreted the decreased expression of inflammatory mediators in *Zfp36aa/aa* lungs as being a direct consequence of increased degradation of pro-inflammatory mRNAs by the non-phosphorylatable form of TTP (TTPaa). This is consistent with properties of TTPaa that have been documented here and elsewhere (Fig. 2) [27, 30–35]. It is also consistent with the fact that pro-inflammatory mRNAs were decreased in *Zfp36aa/aa* lungs 5 days post-inoculation (Fig. 3A), whereas viral loads were not significantly different between *Zfp36+/+* + and *Zfp36aa/aa* mice at that time point (Fig. 5B). It remains possible (but difficult to demonstrate) that some of the illustrated changes in gene expression are actually a result of the accelerated clearance of virus by *Zfp36aa/aa* mice.

In summary, our findings identify phosphorylation of TTP as a critical determinant of pathogenesis in response to a respiratory virus and a putative modulator of the antiviral defence machinery. Pharmacological manipulation of TTP phosphorylation may yield benefits both by reducing harmful inflammation and bolstering antiviral defences.

## Supplementary material

10.1099/jgv.0.002360 Supplementary Material 1.

## Abbreviations

BAL: bronchoalveolar lavage
BM: basement membrane
BMDM: bone marrow-derived macrophage
IAV: influenza A virus
LPS: lipopolysaccharide
MAPK: mitogen-activated protein kinase
PRR: pattern recognition receptor
RIG-I: retinoic acid-inducible gene I
RIPA: radioimmunoprecipitation assay
RT-qPCR: reverse transcription-quantitative polymerase chain reaction
Rux: Ruxolitinib
TLR: toll-like receptor
TPCK: N-p-tosyl-l-phenylalanine chloromethyl ketone
TTP: tristetraprolin
