# Supplementary material for: Mitogen-activated protein kinase p38-dependent phosphorylation of tristetraprolin enhances inflammatory and impairs antiviral responses to influenza virus
Source: J Gen Virol. 2026 Nov 12;107(11):002360. doi: 10.1099/jgv.0.002360 (PMC13759190; doi:10.1099/jgv.0.002360)
Supplement: Supplementary file 1 [file jgv-107-02360-s001.pdf]

# Nair et al. Supplemental Fig. 1

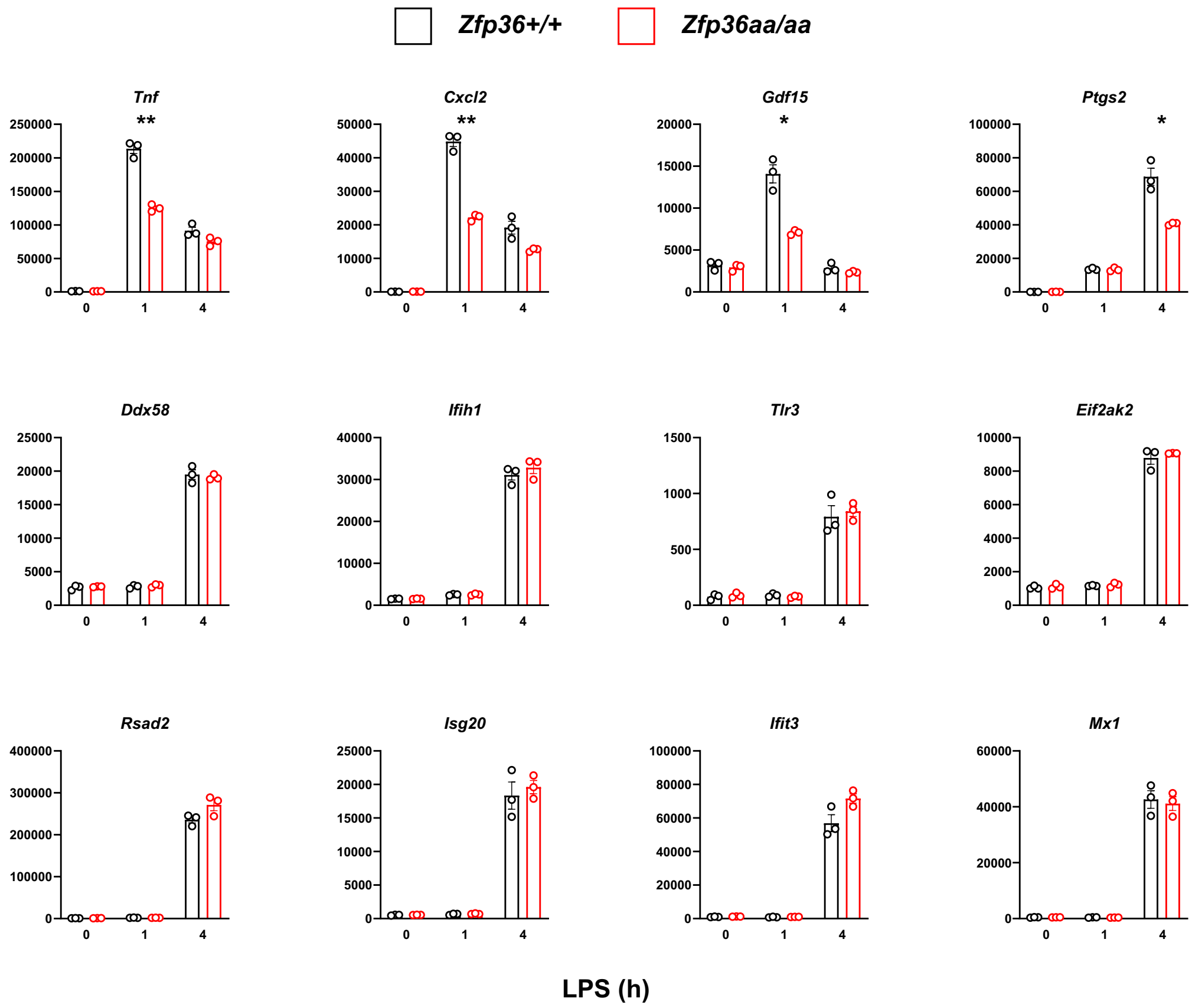


**Supplemental Figure 1. Mutation of the endogenous *Zfp36* locus differentially affects expression of pro-inflammatory and antiviral genes in LPS-activated macrophages.** Wild-type (*Zfp36+/+*) and *Zfp36aa/aa* BMDMs were stimulated with LPS for the indicated times and expression of selected genes was measured by microarray (robust microarray assessment units). *, $p < 0.05$; **, $p < 0.01$ (n=3, two way ANOVA). Data from **31**.
